# Influence of the breathing pattern on the pulmonary function of endurance-trained athletes

**DOI:** 10.1038/s41598-024-51758-5

**Published:** 2024-01-11

**Authors:** Marcin Sikora, Rafał Mikołajczyk, Olga Łakomy, Jakub Karpiński, Aleksandra Żebrowska, Sabina Kostorz-Nosal, Dariusz Jastrzębski

**Affiliations:** 1 Department of Physiological and Medical Sciences, Institute of Healthy Living, The Jerzy Kukuczka Academy of Physical Education, 72A Mikolowska Street, Katowice, Poland; 2 Department of Physiological and Medical Sciences, The Jerzy Kukuczka Academy of Physical Education, 72A Mikolowska Street, Katowice, Poland; 3 Department of Exercise and Sport Performance, Institute of Sport Science, The Jerzy Kukuczka Academy of Physical Education, 72A Mikolowska Street, Katowice, Poland; 4 Department of Lung Diseases and Tuberculosis, Faculty of Medical Sciences in Zabrze, Medical University of Silesia, Zabrze, Poland

**Keywords:** Physiology, Medical research

## Abstract

Proper functioning of the respiratory system is one of the most important determinants of human health. According to current knowledge, the diaphragmatic breathing pattern seems to be the most favourable. However, recent reports indicate that athletes often have dysfunctional breathing patterns, which may be associated with an increased risk of musculoskeletal injuries. The influence of the type of breathing pattern on the mechanical airways in athletes has not been investigated. The aim of the present study was to determine the characteristics and relationships between breathing patterns and respiratory function in athletes. This study included 69 Polish elite endurance athletes (♂40, ♀29) in different sports disciplines and 44 (♂17, ♀27) healthy nonathletes as a control group. All participants underwent pulmonary function tests (spirometry, plethysmography, diffusion capacity for carbon monoxide) with assessment of breathing patterns by the Hi–Lo test. Inspiratory and expiratory resistance (R) and reactance (X) of the respiratory system at a given frequency (5 Hz, 11 Hz, and 19 Hz) were measured by a noninvasive forced oscillation technique. In this study, almost half of the athletes (44.92%) had dysfunctional breathing patterns, although at a lower rate than that in the control group. Diaphragmatic breathing patterns were characterized by higher spirometric, plethysmographic and DLCO values compared to thoracic or abdominal breathing patterns. Similarly, lower inspiratory reactance at 5 Hz (X5%pred.) was observed in the diaphragmatic pattern compared to the thoracic pattern. A diaphragmatic breathing pattern is associated with better pulmonary function test results. However, this study revealed a dysfunctional breathing pattern in almost half of the athletes. These results suggest that the assessment of breathing patterns and the implementation of breathing exercises in athletes are essential to promote proper breathing patterns.

## Introduction

Proper functioning of the respiratory system is one of the most important factors determining the state of human health. During exercise, one of the critical functions of the respiratory system is to adapt the ventilation of the lungs to the increased oxygen demand of the body. Pulmonary function is a determinant of aerobic capacity in athletes. Exercise training has been shown to increase the functional reserve of the respiratory system^1^. Improvements in muscular strength and ventilation in response to endurance training appear to be particularly important in athletes. However, ventilatory work has been found to play a significant role in the cardiovascular response during high-intensity exercise^1^. The main mechanism is the use of inspiratory reserve volume. Exercise training improves endurance and strength of the respiratory muscles in athletes; it also causes a reduction in bronchial resistance and increases lung elasticity and alveolar expansion^2,3^. Studies have also reported increases in lung volume and capacity in response to exercise^2^.

Proper breathing, also known as diaphragmatic breathing, involves synchronized movement of the upper thorax, lower thorax, and abdomen^4^. In addition, proper breathing requires adequate cooperation of the diaphragm and respiratory muscles^5^. Therefore, the key to achieving a proper exercise capacity is to maintain a proper breathing pattern. On the other hand, the presence of dysfunctional breathing patterns in the patient population, such as those with asthma, is well documented and is associated with lower pulmonary function test results^6,7^. To date, the presence of abnormal breathing patterns in athletes and their impact on pulmonary function test results and exercise capacity remains uncertain. Recent reports indicate that dysfunctional breathing patterns are relatively common in athletes and may be associated with an increased risk of musculoskeletal injuries^8^. Additionally, the detection of abnormal breathing patterns in athletes is an important step in the prevention of sports injuries^8^.

The breathing pattern can also significantly influences cardiac autonomic regulation (i.e., cardiorespiratory coupling—CRC), which can directly affect sports performance. So far CRC assessment have been proposed to account for the complex linear and non-linear interactions between respiratory system and heart, as well as their closed loop relationship with feed-back and feed-forward mechanisms^9^. According to Elstad et al.^10^, there are three types of cardiorespiratory interactions that can determine CRC: respiratory sinus arrhythmia; cardioventilatory coupling; and respiratory stroke volume synchronization. CRC coupling appears to provide a great deal of information regarding the physical performance of athletes by depicting it not only quantitatively by measuring maximal oxygen uptake, but also by tracking important changes regarding the blood buffering system and the efficiency of the gas exchange system^11^. The results of the present study point to respiratory pattern as another variable that should be taken into account during CRC analysis.

The forced oscillation technique (FOT) appears to be a potential alternative to traditional methods (spirometry, plethysmography) to assess lung function in athletes. FOT is a non-invasive type of lung function test that allows the assessment of the mechanical properties of both the bronchi and the lung parenchyma^12^. Depending on the frequency of the pressure wave used, impedance provides information on different components of the respiratory system^13^. In contrast to the gold standard for the examination of respiratory function—spirometry—oscillometry is a relatively new test method which has already been used successfully in athletes. Studies have confirmed the high sensitivity of this method in detecting respiratory disorders in athletes^14–16^. FOT has been shown to be more sensitive than spirometry in detecting small peripheral airway diseases. The use of oscillometry in the study of respiratory function in athletes is one of the most important strengths of this work especially as there has been no work to date assessing the effect of breathing pattern on respiratory impedance.

The influence of the type of breathing pattern on pulmonary function test results and respiratory impedance in athletes has not been investigated, which is the main objective of this study.

## Aim

The aim of the present study was to determine the characteristics of and the relationship between breathing patterns and respiratory functions of athletes.

## Methods

### Subjects

This experimental study evaluated the effect of breathing pattern on lung function and mechanical properties of the respiratory system in elite Polish endurance athletes This study included 69 Polish elite endurance athletes (Endurance Athletes Group, EAG) (♂40, ♀29) from different sports disciplines and 44 (♂17, ♀27) healthy nonathlete students from the Academy of Physical Education in Katowice as a control group (CG). Athletes (fourty males and twenty nine females) volunteered for the study. All participants had valid medical examinations and showed no contraindications to participating in the study. They were recruited via contact with the respective coach of the Polish National Sports Associations. The examinations were performed in a certified laboratory of the Institute of Healthy Living at the Academy of Physical Education in Katowice. The athletes participated in a single testing session on a nontraining day. During this session, bronchial mechanical properties, lung function and breathing patterns were analysed. In addition, body composition was assessed using the bioimpedance method (In Body220 Biospace, Inc., Seoul, Korea ISO 9001:2015, EN ISO 13485:2016, EN60601-1, EN60601-1-2). The inclusion criteria for the athlete group were as follows: (1) age over 18 years, (2) training endurance disciplines, (3) training experience of over 6 years, (4) good general health (All athletes were qualified for examination by a sports medicine specialist. Physical examination, blood count, urinalysis, electrocardiography and echocardiography were used as part of the screening process), (5) nonsmoker status, and (6) signing of consent to participate in the research. The inclusion criteria for the control group were as follows: (1) age over 18 years, (2) no exercise discipline, (3) good general health, and (4) signing of consent to participate in the study. The exclusion criteria were as follows: (1) the presence of respiratory diseases; (2) the presence of other diseases, such as neuromuscular diseases, cardiovascular diseases, and obesity, that affect respiratory tract and lung function; and (3) a lack of written consent to participate in the study. The group of athletes included 21 swimmers (Polish national team athletes), 10 ski runners (Polish national team athletes), 15 long-distance runners(Polish national team athletes), 17 soccer players (Polish Premier League players), and 6 triathletes (master class athletes). Only elite endurance trained athletes (long distance runners, and long distance swimmers, triathletes, cross-country skiers and soccer players) with a mean training volume (~ 20 h/week) were included in this study as recommended in the paper: McKinney et al.^17^. The research project was approved of by University Bioethics Committee for Scientific Research at Jerzy Kukuczka Academy of Physical Education-Opinion No 3/2018 of 19 April 2018and conducted in accordance with the Declaration of Helsinki of the World Medical Association,. All research was performed in accordance with relevant guidelines/regulations, and include in their manuscript a statement confirming that informed consent was obtained from all participants and/or their legal guardians. Moreover study participants gave informed consent to the study.

The average sports experience of the athletes was 7.3 ± 1.1 years. The exact characteristics of the tested athletes are presented in Table 1.

**Table 1:** Somatic characteristics of the study groups (endurance athlete group and control group).

Variable	EAG n = 47; 27 M, 20 F	CG n = 44; 17 M, 27 F	F	*p*	η^2^
	Mean ± SD	Mean ± SD			
Age (years)	21.05 ± 1.56	22.42 ± 0.98	0.281	0.755	0.007
Body height (m)	1.76 ± 0.09	1.74 ± 0.05	0.23	0.633	0.003
Body mass (kg)	73.27 ± 10.59	69.62 ± 8.87	0.911	0.342	0.011
BMI (kg/m^2^)	23.38 ± 2.01	22.72 ± 1.42	1.646	0.202	0.019
BSA (m^2^)	1.90 ± 0.18	1.83 ± 0.22	2.763	0.197	0.012

Results of ANOVA analysis of variance, Tukey post hoc test.

BMI, body mass index; BSA, body surface area; F, variation between sample means; p, level of significance; η^2^, effect size.

*p < 0.05 significant differences significant differences in breathing patterns.

The primary outcome was the assessment of lung function in the athletes compared to the control group. The secondary outcome was to evaluate the combined effect of training and breathing pattern.

### Forced oscillation technique

The mechanical properties of the respiratory system were assessed using the noninvasive forced oscillation technique (FOT). FOT was performed with a Resmon Pro Full device (Restech Respiratory Technology SRL, Milan, Italy, ISO 9001:2015, ISO 13485:2016). The measurements were based on the assessment of resistance (R, inspiratory, expiratory), reactance (X, inspiratory, expiratory) at frequencies of 5 Hz and 11 Hz and the differences between the inspiratory and expiratory phases of X at 5 Hz (∆Xrs). The results were expressed as a percentage of the predicted values according to Oostveen et al.^18^. The test was performed during tidal breathing in a sitting position with the cheeks pressed by the examined athlete. The forced oscillation technique was performed first to avoid the likely influence of forced breathing manoeuvres during spirometry on the quality of the results.

The forced oscillation technique (FOT) is a potential alternative to traditional methods (spirometry, plethysmography) that can be used to assess lung function in athletes. FOT is a noninvasive pulmonary function test that allows evaluation of the mechanical properties of both the airways and the lung parenchyma^19^. Depending on the pressure wave frequency used, impedance provides information about different components of the respiratory system^20^. FOT has been shown to be more sensitive than spirometry in detecting small peripheral airway disease. In addition, FOT performed during tidal breathing^12^ can improve the assessment of the respiratory system, which consists of resistance (R) and reactance (X). Resistance reflects the relationship between pressure and the flow of air passing through the airways and is therefore mostly dependent on the airway diameter^12,13^. The use of different frequencies allows airway resistance to be divided into total (R5, at 5 Hz), central (R19, at 19 Hz) and peripheral (R5-R19) resistance. Reactance expresses the ability of the respiratory system to deform^21^. X is determined by the elastic properties that dominate at low frequencies (X5) and the inertial properties of the lung tissue that dominate at high frequencies (X19)^22^.

### Lung function assessment

The athletes' lung function was assessed using PulmOne's MiniBox + (PulmOne Advanced Medical Devices, Ltd, Ra'ananna, Israel ISO 9001:2015), which is a new device approved by the Food and Drug Administration and not yet included in the ATS/ERS guidelines. PulmOne's MiniBox + is a complete pulmonary function testing (PFT) system including cabinless plethysmography, which has been validated as a reliable method to measure absolute lung volumes^23^, as well as spirometry (VC- vital capacity, FVC- forced expiratory volume in one second and FEV1%VC- forced expiratory volume in one second % of vital capacity) and DLCO testing according to guidelines^24–26^. All plethysmographic and spirometric results were expressed in litres and as a percentage of the predicted values.

### Assessment of breathing pattern

**Figure 1:**
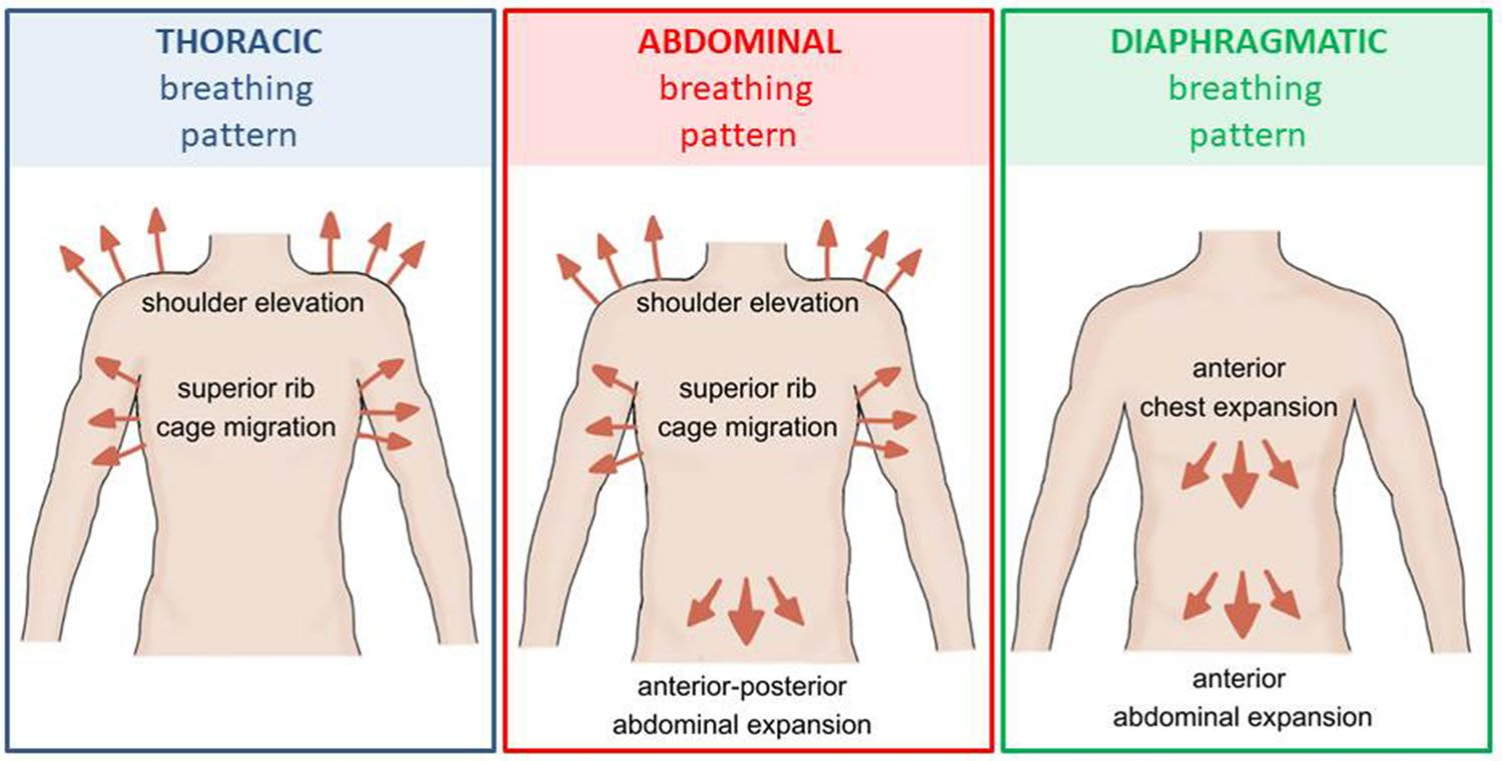
Graphic presentation of the Hi–Lo test.

The breathing pattern was assessed in the standing position by three independent physiotherapists experienced in pulmonary rehabilitation (at the same time) using the Hi–Lo test. Hi–Lo is a manual assessment to determine whether a subject has a normal diaphragmatic breathing pattern or an abnormal pattern. The examiner determines whether thoracic or abdominal movement is dominant during breathing^27^. The reliability of the Hi–Lo test has been reported by others to be acceptable^27,28^. The Hi–Lo test results were used to categorize observations as (a) thoracic-dominant pattern (visible abdominal excursion is absent, but visible superior rib cage migration and shoulder elevation are present), (b) abdominal-only pattern (visible anterior–posterior abdominal expansion is present, but visible superior rib cage migration and shoulder elevation are present), but visible superior rib cage migration and shoulder elevation are present), or (c) diaphragmatic breathing pattern (anterior abdominal expansion followed by anterior chest expansion without superior rib cage migration and shoulder elevation)^29^. The graphical characteristics of the Hi–Lo test are illustrated in Fig. 1.

### Statistical analysis

Microsoft Excel 2007 (Microsoft Corp., Washington, USA) and The Statistics Package v.12 (StatSoft Poland, 13.3) were used for data processing and analysis, and the results are presented as arithmetic means and standard deviations. The magnitudes of differences between the results of the athlete and control groups were expressed as standardized mean differences. The effect size (η2) of breathing patterns and differences between groups were estimated, and the following interpretation was adopted: 0.01–0.06 denoting a weak effect, 0.06–0.14 denoting a medium effect, and over 0.14 denoting a strong effect. The effects of breathing pattern on lung function and respiratory impedance were determined using analysis of variance (ANOVA) (one-way ANOVA and factorial ANOVA). The combined effect of gender and breathing pattern and respiratory impedance was examined using ANOVA analysis of variance(one-way ANOVA and factorial ANOVA). Significant differences between groups and the effects of breathing pattern on lung function and respiratory impedance were analysed using the Tukey post hoc test.

## Results

**Figure 2:**
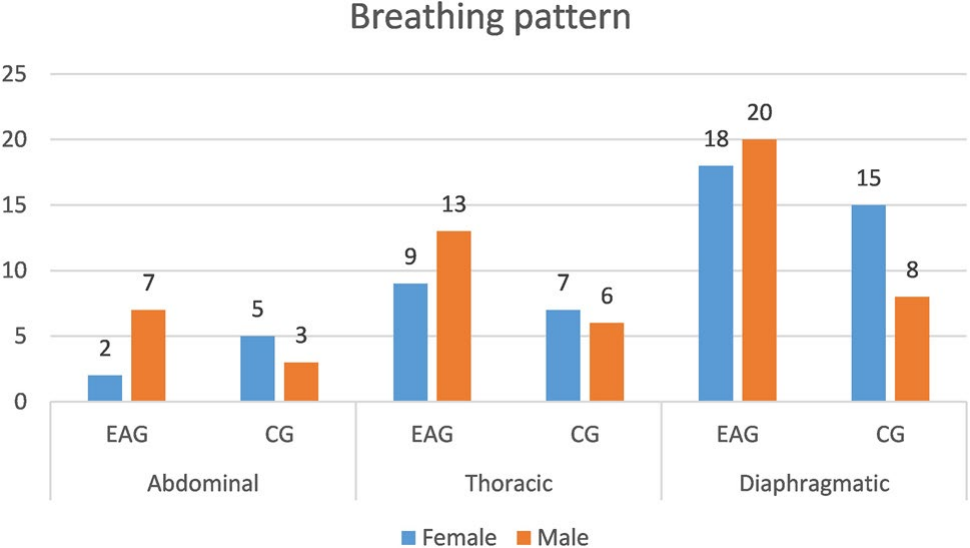
The number of people in the study groups in terms of the occurrence of the breathing pattern.

The tested group of subjects was divided according to their breathing patterns. In the athlete group, 13.04% of the subjects had an abdominal breathing pattern, 31.88% had a thoracic breathing pattern and 55.07% had a diaphragmatic breathing pattern. In the CG group, 18.18% of subjects had an abdominal breathing pattern, 29.54% had a thoracic breathing pattern, and 52.27% had a diaphragmatic breathing pattern. The detailed data are shown in Fig. 2.

The analysis of variance allowed us to observe significantly higher spirometric, plethysmographic and DLCO values for the diaphragmatic breathing pattern compared to the thoracic breathing pattern in the group of athletes for VC (F = 9.77; *p* = 0.000; η^2^ = 0.307), FVC (F = 9.40; *p* = 0.001; η^2^ = 0.299), FEV1 (F = 8.41; *p* = 0.00; η^2^ = 0.276), DLCO mL/min/mmHg (F = 9.48; *p* = 0.000; η^2^ = 0.327), IC (F = 9.777; *p* = 0.001; η^2^ = 0.307), ERV (F = 15.00; *p* = 0.001; η^2^ = 0.312), ERV%PRED (F = 9.770, *p* = 0.017; η^2^ = 0.228), PEF (F = 7.832; *p* = 0.015; η^2^ = 0.262), PIF (F = 10.620; *p* = 0.001; η^2^ = 0.330), TLC (F = 6.486; *p* = 0.003; η^2^ = 0.227), RV/TLC (F = 4.268; *p* = 0.019; η^2^ = 0.162), VA (F = 10.596; *p* = 0.001; η^2^ = 0.352).

Statistical evaluation also revealed higher spirometric values, plethysmographic values and DLCO for the abdominal breathing pattern compared to the thoracic breathing pattern in the group of athletes for VC (F = 9.77; *p* = 0.011; η^2^ = 0.307), FVC (F = 9.40; *p* = 0.011; η^2^ = 0.299), FEV1 (F = 8.41; *p* = 0.006; η^2^ = 0.276) and DLCO mL/min/mmHg (F = 9.48; *p* = 0.030; η^2^ = 0.327), IC (F = 9.777; *p* = 0.011; η^2^ = 0.307), ERV (F = 15.00; *p* = 0.001; η^2^ = 0.312), ERV%PRED (F = 9.770, *p* = 0.002; η^2^ = 0.228), PEF (F = 7.832; *p* = 0.001; η^2^ = 0.262), PIF (F = 10.620; *p* = 0.001; η^2^ = 0.330), TLC (F = 6.486; *p* = 0.044; η^2^ = 0.227), VA (F = 10.596; *p* = 0.038; η^2^ = 0.352).

Differences between diaphragmatic and abdominal breathing patterns in spirometric, plethysmographic, and DLCO values were not statistically significant. Detailed results of the statistical analyses are presented in Table 2. Furthermore, the analysis of variance did not indicate an effect of gender for certain breathing patterns in the athlete study group.

**Table 2:** Breathing pattern differences in spirometry, plethysmography, and DLCO in endurance athletes.

Variables	EAG breathing pattern			F	*p*	Eta2	Post hoc		
	Thoracic ± SD (n = 22, 13♂, 9♀)	Diaphragmatic ± SD (n = 38, 20♂, 18♀)	Abdominal ± SD (n = 9, 7♂, 2♀)				Diaphragmatic versus Thoracic	Abdominal versus Thoracic	Diaphragmatic versus Abdominal
VC (L)	4.05 ± 0.95	5.495 ± 1.13	5.49 ± 0.95	9.777	0.000	0.307	0.001*	0.011*	0.984
IC (L)	2.99 ± 0.79	3.76 ± 0.87	3.31 ± 0.77	6.08	0.003	0.155	0.009*	0.698	0.489
ERV (L)	1.06 ± 0.56	1.73 ± 0.62	2.18 ± 0.45	15.00	0.000	0.312	0.001*	0.001*	0.243
VC (%pred.)	91.213 ± 10.56	106.42 ± 14.10	104.37 ± 9.61	0.075	0.927	0.003	0.991	0.959	0.920
IC%PRED	106.36 ± 16.80	113.40 ± 20.37	97.14 ± 14.24	3.107	0.051	0.086	0.426	0.548	0.160
ERV%PRED	70.37 ± 34.48	96.44 ± 29.83	120.46 ± 22.07	9.770	0.000	0.228	0.017*	0.002*	0.226
FEV1/FVC	85.17 ± 5.06	79.60 ± 18.39	79.59 ± 16.37	2.080	0.136	0.086	0.156	0.991	0.371
FEV1/FVC (%pred.)	97.65 ± 6.01	95.99 ± 7.23	92.71 ± 18.58	0.690	0.507	0.030	0.638	0.962	0.580
FVC (L)	4.85 ± 0.95	6.18 ± 1.18	6.03 ± 0.95	9.400	0.000	0.299	0.001*	0.011*	0.996
FVC (%pred.)	111.27 ± 11.39	113.18 ± 12.75	108.33 ± 9.18	0.037	0.963	0.001	0.997	0.961	0.972
FEV1 (L)	4.14 ± 0.88	5.13 ± 1.01	4.69 ± 0.85	8.415	0.000	0.276	0.001*	0.006*	0.879
FEV1 (%pred.)	107.68 ± 8.91	104.39 ± 8.43	106.62 ± 11.22	0.651	0.526	0.028	0.510	0.960	0.821
PEF (L)	7.59 ± 1.64	9.25 ± 1.93	10.41 ± 1.46	7.832	0.001	0.262	0.015*	0.001*	0.256
PEF% (%pred.)	97.25 ± 11.84	95.82 ± 16.72	105.37 ± 15.03	1.236	0.300	0.053	0.954	0.427	0.275
MEF 50 (l/min)	5.04 ± 1.21	5.21 ± 1.30	6.02 ± 1.87	1.426	0.250	0.060	0.922	0.238	0.334
MEF 50 (%pred.)	103.43 ± 19.28	92.78 ± 20.28	106.00 ± 31.81	1.611	0.211	0.068	0.313	0.961	0.324
PIF (L)	6.25 ± 1.43	8.30 ± 1.42	8.86 ± 2.20	10.620	0.000	0.330	0.001*	0.001*	0.670
TLC (L)	5.75 ± 0.80	7.01 ± 1.33	6.96 ± 1.03	6.486	0.003	0.227	0.003*	0.044*	0.991
TLC (%pred.)	105.87 ± 8.49	101.65 ± 11.72	99.50 ± 10.77	1.201	0.310	0.051	0.443	0.353	0.873
RV (L)	1.14 ± 0.25	1.07 ± 0.55	1.08 ± 0.32	0.126	0.881	0.005	0.876	0.946	0.998
RV (%pred.)	83.81 ± 19.91	69.43 ± 34.13	70.75 ± 20.58	1.331	0.274	0.057	0.266	0.533	0.992
RV/TLC	20.12 ± 4.64	15.08 ± 6.45	15.58 ± 3.44	4.268	0.020	0.162	0.019*	0.146	0.972
RV/TLC (%pred.)	78.81 ± 16.91	66.39 ± 27.81	70.62 ± 15.06	1.403	0.256	0.059	0.227	0.687	0.893
VC (%pred.)	91.213 ± 10.56	106.42 ± 14.10	104.37 ± 9.61	0.075	0.927	0.003	0.991	0.959	0.920
DLco mL/min/mmHg	25.73 ± 5.91	36.31 ± 8.10	34.57 ± 7.43	9.484	0.000	0.327	0.001*	0.030*	0.849
DLco (%pred.)	103.86 ± 11.77	113.40 ± 16.53	109.14 ± 14.65	1.805	0.177	0.084	0.152	0.714	0.788
VA (L)	5.31 ± 1.06	7.34 ± 1.56	6.83 ± 0.86	10.596	0.000	0.352	0.001*	0.038*	0.655
VA%pred	102.66 ± 13.00	110.65 ± 26.68	110.85 ± 7.45	0.748	0.479	0.036	0.492	0.657	0.999
DLco/VA (ml/min/mmHg/L)	4.86 ± 0.58	4.95 ± 0.53	5.05 ± 0.80	0.259	0.773	0.013	0.885	0.769	0.932
DLco/VA%pred	101.13 ± 11.48	97.60 ± 9.52	98.28 ± 14.30	0.452	0.639	0.022	0.623	0.841	0.989
Respiratory frequency (br/min)	13.84 ± 2.52	12.92 ± 2.38	13.33 ± 2.47	0.347	0.739	0.457	0.991	0.838	0.961

Results of ANOVA analysis of variance, Tukey post hoc test All data are presented as the mean ± SD.

VC, vital capacity; IC, inspiratory capacity; ERV, expiratory reserve volume; FVC, forced vital capacity; FEV1, forced expiratory volume in one second; MEF, maximal expiratory flow; PEF, peak expiratory flow; PIF, peak inspiratory flow; TLC, total lung capacity; RV, residual volume; DLco, diffusion lung capacity for carbon monoxide; VA, alveolar volume.

*p < 0.05 significant differences significant differences in breathing patterns.

Analysis of variance revealed significantly lower values of inspiratory reactance at 5 Hz for the diaphragmatic breathing pattern compared to the thoracic breathing pattern (inspiratory %pred—96.41% vs. 128.72%). Detailed data on the differences in respiratory impedance with respect to the observed breathing patterns are presented in Table 3.

**Table 3:** Differences in respiratory impedance between diaphragmatic, thoracic, and abdominal breathing patterns in endurance athletes.

Variables	EAG breathing pattern			F	*p*	Eta2	Post hoc		
	Thoracic ± SD (n = 22. 13♂. 9♀))	Diaphragmatic ± SD (n = 38. 20♂. 18♀)	Abdominal ± SD (n = 9. 7♂. 2♀)				Diaphragmatic versus thoracic	Abdominal versus thoracic	Diaphragmatic versus abdominal
Rinsp5 (%pred.)	123.83 ± 28.41	108.15 ± 26.89	117.63 ± 31.81	2.165	0.122	0.061	0.170	0.889	0.762
Rexp5 (%pred.)	138.44 ± 32.19	121.30 ± 33.83	135.80 ± 39.64	1.988	0.144	0.056	0.225	0.985	0.640
X_insp_5 (%pred.)	128.72 ± 44.87	96.41 ± 29.10	91.35 ± 22.67	7.125	0.001	0.177	0.007*	0.061	0.947
X_exp_5 (%pred.)	105.52 ± 39.22	82.50 ± 38.19	84.58 ± 49.01	2.409	0.097	0.068	0.144	0.510	0.993
R_insp_11 (%pred.)	113.79 ± 26.03	102.81 ± 24.14	111.99 ± 38.37	1.309	0.276	0.038	0.369	0.988	0.749
R_exp_11 (%pred.)	134.87 ± 31.08	120.96 ± 32.56	133.98 ± 40.77	1.446	0.242	0.041	0.352	0.998	0.684
X_insp_11 (%pred.)	222.78 ± 570.40	261.89 ± 709.41	1193.80 ± 2146.00	3.723	0.029	0.101	0.990	0.093	0.112
X_exp_11 (%pred.)	275.79 ± 442.44	226.64 ± 451.37	1071.80 ± 2189.11	3.556	0.034	0.097	0.980	0.135	0.106
R_insp_19 (%pred.)	111.32 ± 24.92	100.46 ± 20.88	110.05 ± 36.30	1.572	0.215	0.045	0.312	0.993	0.685
R_exp_19 (%pred.)	122.89 ± 24.87	110.64 ± 26.32	123.37 ± 34.84	1.791	0.174	0.051	0.296	0.999	0.580
∆Xrs	-0.08 ± 0.21	-0.1 ± 0.19	-0.09 ± 0.23	1	0.97	0.005	0.990	0.982	0.976

Results of ANOVA analysis of variance, Tukey post hoc test. All data are presented as the mean ± SD.

R, resistance; X, reactance; insp, inspiratory; exp, expiratory; ∆Xrs, the differences between inspiratory and expiratory phases of X at 5 Hz.

*p < 0.05 significant differences significant differences in breathing patterns.

In addition, the factorial analysis of variance also indicated the combined effect of regular training and breathing pattern on spirometric values. Compared to the control group, athletes had significantly higher values for VC (diaphragmatic breathing pattern, *p* = 0.007), FVC (diaphragmatic breathing pattern, *p* = 0.001), FEV1 (diaphragmatic breathing pattern, *p* = 0.001), and VC %pred (all breathing patterns abdominal *p* = 0.023, thoracic *p* = 0.001, and diaphragmatic *p* = 0.027). No combined effect of training and breathing pattern on respiratory impedance was observed. Detailed results of the analysis are presented in Table 4.

**Table 4:** Combined effect of training and breathing pattern on spirometric values and respiratory impedance.

Variables	EAG breathing pattern			CG breathing pattern			F	*p*	Eta2	Post hoc (CG vs. EAG)		
	Thoracic ± SD (n = 22, 13♂, 9♀)) SD	Diaphragmatic (n = 38, 20♂, 18♀) SD	Abdominal (n = 9, 7♂, 2♀) SD	Thoracic (n = 13, 6♂, 7♀)) SD	Diaphragmatic (n = 23, 8♂, 15♀) SD	Abdominal (n = 8, 3♂, 5♀) SD				Abdominal versus abdominal	Thoracic versus thoracic	Diaphragmatic versus diaphragmatic
VC (L)	4.05 ± 0.95	5.49 ± 1.13	5.49 ± 0.95	3.46 ± 0.72	4.47 ± 0.90	4.13 ± 0.89	0.934	0.396	0.017	0.071	0.634	0.007*
IC (L)	2.99 ± 0.79	3.76 ± 0.87	3.31 ± 0.77	2.90 ± 0.94	3.17 ± 0.75	3.11 ± 0.94	1.017	0.365	0.018	0.996	0.999	0.167
ERV (L)	1.059 ± 0.56	1.73 ± 0.62	2.18 ± 0.45	0.56 ± 0.41	1.30 ± 0.55	1.03 ± 0.37	2.914	0.058	0.051	0.001*	0.191	0.086
VC %pred	91.213 ± 10.56	106.42 ± 14.10	104.37 ± 9.61	70.94 ± 9.03	96.64 ± 4.98	87.52 ± 3.97	2.706	0.071	0.048	0.023*	0.001*	0.027*
IC %pred	106.36 ± 16.80	113.41 ± 20.37	97.14 ± 14.25	90.66 ± 17.65	108.08 ± 16.37	101.04 ± 13.78	1.835	0.164	0.033	0.997	0.224	0.912
ERV % pred	70.38 ± 34.48	96.44 ± 29.83	120.47 ± 22.08	36.87 ± 28.52	81.71 ± 26.67	67.96 ± 28.99	3.017	0.053	0.053	0.007*	0.050	0.539
FEV1/FVC	85.17 ± 5.06	79.60 ± 18.39	79.59 ± 16.37	84.30 ± 0.85	84.30 ± 0.93	84.00 ± 1.07	0.60	0.548	0.011	0.976	0.999	0.763
FEV1/FVC%pred	97.65 ± 6.01	95.99 ± 7.23	92.71 ± 18.58	96.46 ± 8.14	96.47 ± 7.25	96.25 ± 8.33	0.42	0.656	0.007	0.962	0.999	0.999
FVC (L)	4.85 ± 0.95	6.18 ± 1.18	6.03 ± 0.95	4.83 ± 1.18	4.74 ± 1.12	4.85 ± 0.93	5.46	0.026	0.158	0.262	1.000	0.001*
FVC% pred	111.27 ± 11.39	113.18 ± 12.75	108.33 ± 9.18	102.61 ± 7.04	105.39 ± 10.23	105.75 ± 12.94	8.918	0.005	0.235	0.997	0.368	0.181
FEV1 (L)	4.14 ± 0.88	5.13 ± 1.01	4.69 ± 0.85	3.94 ± 0.87	3.80 ± 1.07	3.68 ± 1.20	3.51	0.033	0.061	0.312	0.996	0.001*
PEF (L)	7.59 ± 1.64	9.25 ± 1.93	10.41 ± 1.46	7.59 ± 1.64	7.67 ± 1.87	7.99 ± 1.79	2.84	0.063	0.062	0.082	1.000	0.037*
PEF%pred	97.25 ± 11.84	95.82 ± 16.72	105.37 ± 22.81	86.61 ± 11.09	89.95 ± 9.11	91.25 ± 4.30	0.75	0.472	0.017	0.221	0.262	0.605
MEF 50	5.04 ± 1.21	5.21 ± 1.30	6.02 ± 1.87	4.62 ± 1.34	4.54 ± 1.44	4.29 ± 0.95	1.28	0.282	0.029	0.119	0.969	0.547
MEF 50%pred	103.43 ± 19.28	92.78 ± 20.28	106.00 ± 31.81	87.92 ± 25.32	88.47 ± 21.94	83.62 ± 8.67	1.23	0.295	0.028	0.323	0.464	0.984
Rinsp5 (%pred.)	123.84 ± 28.41	108.15 ± 26.89	117.63 ± 31.81	108.72 ± 17.28	120.28 ± 28.92	115.55 ± 28.93	2.572	0.081	0.045	0.999	0.730	0.672
Rexp5 (%pred.)	138.45 ± 32.19	121.31 ± 33.83	135.80 ± 39.65	123.45 ± 20.57	133.65 ± 41.56	124.14 ± 33.43	1.928	0.150	0.034	0.984	0.877	0.829
X_insp_5 (%pred.)	128.72 ± 44.87	96.41 ± 29.10	91.36 ± 22.67	143.73 ± 40.89	125.92 ± 38.81	122.76 ± 42.26	0.476	0.622	0.008	0.524	0.901	0.077
X_exp_5 (%pred.)	105.53 ± 39.22	82.51 ± 38.19	84.59 ± 49.01	120.72 ± 28.40	106.59 ± 38.15	94.51 ± 40.96	0.281	0.754	0.005	0.995	0.915	0.285
R_insp_11 (%pred.)	113.79 ± 26.03	102.81 ± 24.14	111.99 ± 38.38	94.59 ± 16.42	111.05 ± 29.55	112.25 ± 31.19	2.74	0.069	0.048	1.000	0.452	0.902
R_exp_11 (%pred.)	134.87 ± 31.08	120.96 ± 32.56	133.98 ± 40.77	117.48 ± 22.06	133.14 ± 39.93	127.48 ± 37.14	2.07	0.131	0.037	0.998	0.781	0.827
X_insp_11 (%pred.)	222.78 ± 570.40	261.89 ± 709.41	1193.80 ± 2146.01	214.86 ± 280.53	320.13 ± 939.84	256.87 ± 300.52	2.17	0.118	0.039	0.283	1.000	0.999
X_exp_11 (%pred.)	275.79 ± 442.44	226.64 ± 451.37	1071.80 ± 2189.11	147.88 ± 232.45	170.79 ± 201.94	195.92 ± 284.41	2.33	0.101	0.041	0.130	0.997	0.999
R_insp_19 (%pred.)	111.32 ± 24.92	100.46 ± 20.88	110.05 ± 36.30	90.35 ± 18.84	105.93 ± 27.00	109.35 ± 28.09	3.00	0.053	0.053	1.000	0.265	0.975
R_exp_19 (%pred.)	122.89 ± 24.87	110.64 ± 26.32	123.37 ± 34.85	104.10 ± 20.57	115.87 ± 31.61	116.26 ± 29.47	2.00	0.139	0.036	0.995	0.511	0.987
∆Xrs	-0.08 ± 0.21	-0.1 ± 0.19	-0.09 ± 0.23	-0,10 ± 0.20	-0.12 ± 0.21	-0.10 ± 0.21	0.92	0.983	0.003	0.998	0.999	1.000

Results of ANOVA analysis of variance, Tukey post hoc test. All data are presented as the mean ± SD.

VC, vital capacity; IC, inspiratory capacity; ERV, expiratory reserve volume; FVC, forced vital capacity; FEV1, forced expiratory volume in one second; MEF, maximal expiratory flow; PEF, peak expiratory flow; R, resistance; X, reactance; insp, inspiratory; exp, expiratory; ∆Xrs, the differences between inspiratory and expiratory phases of X at 5 Hz.

*p < 0.05 significant differences significant differences in breathing patterns.

## Discussion

One of the main novelties of this work is the use of the technique of forced oscillations to study the differences between the breathing patterns used by athletes. Interestingly, in the group of athletes, significantly lower reactance values (5 Hz %pred) were observed in subjects using the diaphragmatic breathing pattern compared to the thoracic pattern. Reactance is determined by the elastic properties of the lung tissue, which dominate at low frequencies (X5), and the inertial properties of the lung tissue, which dominate at high frequencies (X19)^22^. Nevertheless, X5 is also lower in the presence of heterogeneity in airway diameter distribution, which can be present in obstructive lung disease, most commonly when small airways collapse. Regardless of the mechanism, such a decrease in reactance in the group of athletes underlines the advantage of the diaphragmatic breathing pattern, expressed in the improvement of lung function. However, it should be remembered that the observed results are still within the normal range for healthy people, and we only present the tendency to change the elasticity of the lung parenchyma or the communication between different parts of the lung under the influence of training. These results are in agreement with previously published studies that indicate an increase in lung elasticity as a result of physical training^13,21^. Thus far, scientific reports only explain the occurrence of obstructive disorders in athletes, especially winter sports activity, which puts athletes at risk of asthma and exercise-induced bronchoconstriction. This is thought to be the result of repeated dehydration of the small airways when large volumes of cold air are inhaled^30^. Exercise-induced bronchospasm, which occurs after vigorous exercise, may be triggered by intense exercise, cold dry environments, chronic asthma, or a variety of air pollutants^31^. The scientific literature indicates that physical exercise increases the endurance and strength of the respiratory muscles; it also causes a reduction in bronchial resistance and increases the elasticity of the lung tissue, allowing free expansion of the alveoli^32^. However, previous studies were mainly based on the analysis of spirometric values, the results of which can only suggest an increase in the elasticity of the lung tissue. The main advantage of this research is the use of the forced oscillation technique, which is considered a much more sensitive research tool. The significantly lower X5 (%pred.) is in line with other better pulmonary function test results observed in athletes and confirms the positive effect of the diaphragmatic pattern on the functioning of the respiratory system.

Another important observation of this study is the high prevalence of dysfunctional/unfavourable breathing patterns both in the group of endurance athletes (44.92%) and in the control group (47.73%) of physically active young healthy people. According to our results, the diaphragmatic breathing pattern is characterized by better lung function test results. In addition, we found that athletes had higher spirometric and plethysmographic results than the control group. At the same time, no effect of physical activity was observed on oscillometric results, which seems to be independent of respiratory muscle and diaphragm function. These results confirm the effect of training on the diaphragm only observed in pulmonary function tests requiring forced expiratory manoeuvres and therefore demonstrate the advantage of the diaphragmatic breathing pattern over the others. A particularly common but undesirable pattern, especially in athletes, is the thoracic one. Endurance athletes have higher VC, FEV1, FVC and VC due to the effect of training on the diaphragm and respiratory muscles.

In addition, studies indicate a lower efficiency of the respiratory system in subjects with a confirmed dysfunctional breathing pattern. Scientific publications indicate a high prevalence of dysfunctional breathing patterns (nondiaphragmatic patterns) in the population of healthy, physically active adults^29^. In addition, lower physical activity has been observed in subjects with confirmed dysfunctional breathing^27^. In the work of Shimozawa et al.^8^, the prevalence of dysfunctional breathing patterns was even higher (90.6%). Despite the lower number of dysfunctional breathing patterns observed, the prevalence of dysfunctional breathing patterns in the athletic population cannot be ignored^8^, as biomechanical breathing patterns have been associated with various musculoskeletal and psychological conditions and the health status of individuals^33–35^. In addition, other reports indicate that dysfunctional breathing strategies influence functional movement patterns^36^. In addition, a study by Shimozawa et al.^8^ suggests that dysfunctional breathing patterns may also increase the risk of musculoskeletal injury during exercise. Although dysfunctional breathing patterns have been observed less frequently in athletes than in the general population, their high prevalence remains a significant clinical problem, and therefore, an assessment of breathing patterns may be necessary to prevent injury in the athletic population. Therefore, it seems necessary to include breathing exercises in athletic training that would allow the formation of a correct breathing pattern. Previous studies suggested that including respiratory muscle training could optimize the respiratory pattern and aerobic and aerobic capacity in athletes^37–39^. Strengthening the inspiratory muscles increases respiratory performance, endurance and reduces blood lactate concentration. Contrary, fatigue in respiratory muscle during exercise limits their optimal physiological function and decreases oxygen supply to working muscle^40^.

Another important finding of our study was the demonstration of no differences in the incidence of different breathing patterns between the sexes. However, the literature indicates that the thoracic breathing pattern is more common in women^41,42^. This seems to be related to respiratory exercises. Positive changes in breathing patterns induced by exercise have been reported in COPD patients^43^. Unfortunately, no similar work has been found in athletes, even if a change in the dysfunctional breathing pattern seems possible. In addition, importantly, in highly trained endurance athletes, changes in the training load during the training macrocycle do not have any effect on the change in the breathing pattern^44^.

This study also confirms the significant influence of the breathing pattern on spirometric results. Significantly higher spirometric values were observed in athletes using the diaphragmatic breathing pattern compared to the thoracic pattern. In addition, a significantly higher RV/TLC ratio and a lower diffusion for carbon monoxide (DLCO) value were observed in subjects using the thoracic breathing pattern in relation to the diaphragmatic breathing pattern. These results confirm the negative impact of dysfunctional breathing patterns on lung function. This condition appears to be particularly important in the athletic population due to the possibility of decreased physical performance. It is generally accepted that elite athletes and physically active individuals tend to have higher spirometric values, which are influenced by many factors, such as strength, agility, power, speed, and cardiovascular endurance^32,45–47^. The reason why athletes have higher spirometric lung volumes than sedentary controls is mostly due to respiratory adaptations to exercise^46,48,49^. Interestingly, there are also reports that the introduction of additional inspiratory muscle training can enhance the training effects on lung function in athletes and at the same time improve their performance^50,51^. These results are consistent with our report, which showed higher spirometric and plethysmographic results in the group of athletes. However, no effect of training on lung function was observed in the oscillometric results. These results confirm the higher sensitivity of FOT compared to spirometry, which excludes subject-related factors, thus confirming its objectivity.

Recent reports indicate a significant effect of endurance training on spirometric values in athletes. Hacket^1^indicated that endurance training experience has yielded greater performance in FVC, FEV1, VC and MVV. Similar observations were confirmed by the reports of Durmic et al.^13^ and Lazovic et al.^32^. The presence of improved lung function is likely the result of training adaptations to greater and prolonged ventilation to meet the gas exchange demands of exercise^1^. These high demands on the respiratory system during endurance training and events are reflected in the occurrence of hypoxemia in some athletes^52^. In addition, other studies have shown that endurance athletes have higher FVC and FEV 1 values but lower FEV 1/FVC values than the sedentary population^53^.

Our report shows a higher diffusion value for carbon monoxide in athletes breathing with the diaphragmatic breathing pattern compared to the others. To date, no such relationship has been reported in the scientific literature. Nevertheless, the results we obtained are consistent with the basis of diffusion measurement, as it is well documented that exercise contributes to an increase in DLCO^54^. During exercise, the surface area of the functioning alveoli increases and therefore has greater contact with the pulmonary capillaries. Not surprisingly, diaphragmatic breathing patterns associated with better lung function are characterized by higher DLCO results. The reports indicate that the DLCO may double in individuals who exercise regularly in proportion to the increase in cardiac output^55^, which may explain the fact that body fat% has no effect on the variability of DLCO in elite athletes^49^. Furthermore, there is a report indicating that DLCO is positively correlated with lean body mass^56^.

In this study, a significant effect of the breathing pattern on lung function was observed. According to our results, a dysfunctional breathing pattern was associated with lower lung function test results. To date, there are no reports describing the influence of breathing patterns on lung function in a group of endurance athletes. However, the effect of endurance training on changes in breathing patterns during intense exercise has been analysed, but no significant changes were observed^44^.

### Study limitations

The main limitations of this study are the small size of the group of athletes tested and the lack of assessment of exercise capacity as a determinant of lung function and airway mechanical properties. Despite the relatively small size of the group, the results based on oscillometry seem to be reliable, if only because of the sensitivity of the device. However, this is a pioneering study showing the impact of breathing pattern on lung function tests. Future studies are therefore needed to establish the implementation of breathing pattern assessment in the individual training of subjects.

## Conclusions

A diaphragmatic breathing pattern is characterized by better lung function test results. However, almost half of the athletes in this study had a dysfunctional breathing pattern. These results suggest that assessment of the breathing pattern may be necessary to identify dysfunctional breathing patterns in athletes. It may also be important to incorporate breathing exercises into an athlete's training to help develop a proper breathing pattern and thus better exercise performance.

## Data Availability

Data and publication materials are available upon request to the corresponding author.
